# Aberrant Hypermethylation-Mediated Suppression of PYCARD Is Extremely Frequent in Prostate Cancer with Gleason Score ≥ 7

**DOI:** 10.1155/2021/8858905

**Published:** 2021-02-04

**Authors:** Toshiya Miyauchi, Masahiro Takahashi, Koji Mitsuzuka, Yuriko Saiki, Teppei Okubo, Paula M. Vertino, Akiteru Goto, Yoichi Arai, Akira Horii, Shinichi Fukushige

**Affiliations:** ^1^Division of Pathology, Tohoku University School of Medicine, Sendai, Miyagi 980-8575, Japan; ^2^Department of Cellular and Organ Pathology, Graduate School of Medicine, Akita University, Akita 010-0825, Japan; ^3^Department of Urology, Tohoku University School of Medicine, Sendai, Miyagi 980-8574, Japan; ^4^Departments of Biomedical Genetics and Pathology and Laboratory Medicine, University of Rochester School of Medicine and Dentistry, Rochester, NY 14642, USA; ^5^Center for Regulatory Epigenome and Diseases, Tohoku University School of Medicine, Sendai, Miyagi 980-8575, Japan

## Abstract

Epigenetic gene silencing by aberrant DNA methylation leads to loss of key cellular pathways in tumorigenesis. In order to analyze the effects of DNA methylation on prostate cancer, we established LNCaP-derived human prostate cancer cells that can pharmacologically induce global reactivation of hypermethylated genes by the methyl-CpG targeted transcriptional activation (MeTA) method. The MeTA suppressed the growth of LNCaP-derived cells and induced apoptosis. Microarray analysis indicated that *PYCARD* (PYD and CARD domain containing) encoding an apoptosis-inducing factor was upregulated by 65-fold or more after treatment with MeTA. We analyzed DNA methylation statuses using 50 microdissected primary prostate cancer tissues and found an extremely high frequency of tumor-specific promoter hypermethylation of *PYCARD* (90%, 45/50). Moreover, DNA methylation status was significantly associated with Gleason score (*P* = 0.0063); the frequency of tumor-specific hypermethylation was 96% (44/46) in tumors with Gleason score ≥ 7, whereas that in tumors with Gleason score 6 was 25% (1/4). Immunohistochemical analyses using these 50 cases indicated that only 8% (4/50) of cancerous tissues expressed PYCARD, whereas 80% (40/50) of corresponding normal prostate epithelial and/or basal cells expressed PYCARD. In addition, there was no relationship between PYCARD immunostaining and the Gleason score in cancerous tissue and surrounding normal tissue. Inducible expression of *PYCARD* inhibited cell proliferation by induction of apoptosis. These results suggest that aberrant methylation of *PYCARD* is a distinctive feature of prostate cancers with Gleason score ≥ 7 and may play an important role in escaping from apoptosis in prostatic tumorigenesis.

## 1. Introduction

Cancer cells acquire their hallmarks of malignancy through successive aberrations in the controlling systems for growth suppression, proliferative signaling, cellular energetics, cell death, genomic stability, angiogenesis, invasion and metastasis, response to tumor promoting inflammation, immortalization, and response to immune system [1]. Cancer cells acquire these hallmarks and stably transmit them to their daughter cells, mainly through genetic and epigenetic alterations [2]. DNA methylation and particular forms of histone modification are two major mechanisms in epigenetic transcriptional control. Among these, aberrant DNA methylation of either hypermethylation or hypomethylation in the promoter regions of genes is one of the most well-defined epigenetic changes in cancer cells and is associated with inappropriate expression of certain genes [3, 4]. A number of genes with common aberrant hypermethylation mainly at the promoter region have been described so far, but the relationships between each promoter hypermethylation and its contribution to tumorigenesis remain to be elucidated.

In men, prostate cancer is the second most frequent cancer and the fifth leading cause of cancer death worldwide [5]. Intense studies have explored the molecular bases of primary prostate cancer and have identified multiple genetic and epigenetic alterations [6]. The most frequent alteration in prostate cancer is fusion of the 5′ untranslated region of *TMPRSS2*, an androgen-regulated serine protease gene, with the oncogenic ETS family transcription factors of either *ERG* or *ETV1* [7], and the most frequently mutated genes are *SPOP*, *TP53*, *FOXA1*, and *PTEN* [8]. Although epigenetic changes in prostate cancer have been explored extensively, the significance of their alterations remains elusive. Glutathione S-transferase pi 1 (*GSTP1*) is the most-characterized hypermethylated gene in prostate cancer [9]; it encodes an enzyme that detoxifies reactive electrophilic intermediates [10]. Promoter hypermethylation-mediated silencing of *GSTP1* is only detected in intraepithelial neoplasia, prostatic adenocarcinoma, and fluids (plasma, serum, ejaculate, and urine) of patients with prostate cancer but is never detected in benign epithelium [9, 11–13]. Although *GSTP1* has such promising features as a cancer-specific biomarker, it has not yet been applied in a clinical setting; although the specificity of *GSTP1* promoter hypermethylation is much higher than that of prostate-specific antigen (PSA), the sensitivity of *GSTP1* is lower than that of PSA [14]. In addition, *GSTP1* did not function as a tumor suppressor gene when using LNCaP prostate cancer cells *in vitro* and *in vivo* [15]. Thus, identification of a novel biomarker with much higher specificity and sensitivity for early detection and prognosis of prostate cancer is still awaited.

In the present study, we first attempted to elucidate the biological significance of epigenetic silencing in prostate cancer and then detected hypermethylated genes that play important roles in prostatic tumorigenesis by applying our previously developed method termed methyl-CpG targeted transcriptional activation (MeTA) [16–19]. MeTA can globally reactivate hypermethylated genes including transcriptionally silenced cancer-related genes. The cell line LNCaP was used as a representative of prostate cancer. Because microarray coupled with MeTA (MeTA-array) can search for hypermethylated genes by utilizing a quite different mechanism from DNA demethylating agent-based method, it enables us to unveil yet-to-be-discovered hypermethylated genes.

In the present study, we identified *PYCARD* as a DNA hypermethylation-mediated silencing gene that plays a key role in cell growth suppression, mainly by induction of apoptosis. Aberrant promoter hypermethylation of *PYCARD* along with suppressed protein expression was observed in a stage-specific manner in the great majority of prostate cancer specimens.

## 2. Materials and Methods

### 2.1. Strains and Plasmids

*E. coli* strain DH5*α*F′ was used to propagate all the plasmids. pcDNA6/TR plasmid(Invitrogen, Carlsbad, CA, USA) was used to establish cell lines that stably express the Tet repressor. To allow tetracycline-regulated expression, we used three constructs. One contains a 0.9 kb fragment of NF*κ*B (AD)-MBD with a 3xFLAG tag at the N-terminus [16] that was cloned into the *Eco*RI/*Xho*I sites of pcDNA4/TO/myc-His vector (Invitrogen) and named as pcDNA4/TO/NF*κ*B (AD)-MBD. The other two constructs contain cDNA fragments corresponding to the entire coding regions of *PYCARD* variants 1 and 2, a 0.61 kb fragment of *PYCARDv1* and a 0.56 kb fragment of *PYCARDv2*. These two fragments were cloned into the *Hin*dIII/*Xba*I sites of pcDNA4/TO/myc-His vector and named as pcDNA4/TO/PYCARDv1 and pcDNA4/TO/PYCARDv2, respectively. We PCR amplified these two variant cDNA fragments using the pooled human cDNA mix [20] as the template. Nucleotide sequences of the primers used for cDNA cloning are described in Table S1.

### 2.2. Cell Culture, Transfection, and Immunoblotting

Human prostate cancer cell line LNCaP and normal prostate epithelial cell line RWPE-1 were purchased from American Type Culture Collection (ATCC, Manassas, VA, USA). LNCaP cells were grown in RPMI-1640 medium (Sigma, St. Louis, MO, USA) supplemented with 10% fetal bovine serum (Invitrogen), and RWPE-1 cells were grown in keratinocyte serum-free medium supplemented with bovine pituitary extract and human recombinant epidermal growth factor (Invitrogen). Tet repressor cells were established by stably transfected pcDNA6/TR plasmid in LNCaP, as described previously [20], and were named as TR9 and TR15, respectively. Then, pcDNA4/TO/NF*κ*B(AD)-MBD was stably transfected into TR9 or TR15 cells to obtain TR9_MeTA14 and TR15_MeTA5 cells. pcDNA4/TO/PYCARDv1 or pcDNA4/TO/PYCARDv2 was transfected into TR15 cells to establish stable transfectants of tetracycline-regulated PYCARD-expressing cells. Empty pcDNA4/TO/myc-His vector was also stably transfected into TR15 cells to establish the control TR15_Vec cells. All these cell lines were maintained in RPMI-1640 medium containing 7.5 *μ*g/ml blasticidin and 500 *μ*g/ml zeocin. Immunoblotting analyses were performed as described previously [21]. An antibody that specifically recognizes FLAG (F 1804; 1 : 1000, Sigma-Aldrich), PYCARD (D086-3; 1 : 1000, MBL, Nagoya, Japan), or *β*-actin (A-5441; 1 : 3000, Sigma-Aldrich) was used.

### 2.3. Reverse Transcription-PCR (RT-PCR)

Total RNAs were extracted from harvested cell pellets by RNeasy Mini Kit (Qiagen, Valencia, CA, USA). Each aliquot of 2 *μ*g of total RNA was reverse transcribed, and single-stranded cDNA was synthesized using a High-Capacity cDNA Reverse Transcription Kit (Applied Biosystems, Foster City, CA, USA). RT-PCR amplifications using intron-spanning primers were performed as described [22], and *B2M* was used as the internal control [23]. PCR products were analyzed on 3% agarose gels, and the bands were visualized by ethidium bromide staining. Primers were purchased from Integrated DNA Technologies (Coralville, IA, USA), and their nucleotide sequences are shown in Table S1.

### 2.4. Cell Proliferation Assay

Cells were seeded in 24-well dishes at a density of 3 × 10^3^ cells per well at day -1, and the assay started at day 0. After the first assay, cells were treated with or without 1 *μ*g/ml tetracycline for 2, 4, and 6 days. Cell viability was determined every other day by using alamarBlue (Invitrogen) as described previously [24]. Experiments were performed in quadruplicate and repeated three times.

### 2.5. Cell Cycle Analysis

Cells were seeded in 10 cm dishes and were treated with or without 1 *μ*g/ml tetracycline for 2, 4, and 6 days. Tetracycline-containing medium was replaced every other day. Cell cycle distributions of samples were determined by flow cytometric analysis (FACSCanto II; BD Biosciences, San Jose, CA, USA) as described previously [25].

### 2.6. Terminal Deoxynucleotidyl Transferase dUTP Nick End Labeling (TUNEL) Assay

Apoptotic cells were quantified using an apoptosis *in situ* detection kit (Wako, Osaka, Japan). Cells were permeabilized with 0.1% sodium citrate and 0.1% Triton X-100 on ice for 2 min. DNAs were labeled with terminal deoxynucleotidyl transferase, and the intrinsic peroxidase was inactivated with 3% H_2_O_2_. Finally, cells were incubated with a peroxidase-conjugated antibody. Peroxidase activity in each sample was visualized by the application of DAB (3, 3′-diaminobenzidine tetrahydrochloride, Sigma).

### 2.7. Microarray Analyses

Microarray analyses were performed according to methods described previously [18]. Total RNAs from TR9_MeTA14 and TR15_MeTA5 cells with or without tetracycline treatments for 4 days were prepared, and Cy3-labeled cRNA was hybridized to an Agilent whole human genome microarray (4 × 44K). A cut-off value of 2-fold upregulation was employed for selection of the genes.

### 2.8. Sodium Bisulfite Sequencing

We carried out sodium bisulfite modifications of genomic DNAs from RWPE-1 or TR9_MeTA14 and TR15_MeTA5 cells with or without tetracycline treatment for 4 days using EpiTect Bisulfite Kit (Qiagen) and sequenced the promoter regions of *PYCARD* and *TNFRSF25* genes on an ABI PRISM 310 sequencer with BigDye Terminator v3.1 Cycle Sequencing Kit (Applied Biosystems). Primers used for sodium bisulfite sequencing analyses are described in Table S1.

### 2.9. Immunofluorescence Staining

TR15_PYCARD cells with or without tetracycline treatment for two days were washed with PBS, fixed, and incubated with rabbit anti-human TMS1 antibody (EU107, 1 : 1000) [26]. Indirect detection of primary antibodies was achieved by 60 min incubation with 1 : 1000 diluted secondary antibodies: Cy3-labeled goat anti-rabbit IgG (Abcam ab6939). Cells were stained for 10 min with 0.5 *μ*g/ml 4′, 6′-diamidino-2-phenylindole (DAPI). Images were acquired as described previously [20].

### 2.10. Tissue Specimens

A total of 50 prostate cancer tissues obtained from patients who had radical prostatectomy at Tohoku University Hospital (Sendai, Miyagi, Japan) during the period from 2008 to 2011 were analyzed. All of them were Asians. None of them had received radiotherapy, chemotherapy, or androgen deprivation treatment prior to surgery. Biochemical recurrence was defined as postoperative PSA levels ≥ 0.2 ng/ml after a nadir PSA level < 0.2 ng/ml. Tissue specimens were formalin-fixed, paraffin-embedded (FFPE), and evaluated by hematoxylin and eosin (HE) staining. Histopathological diagnosis was verified by a pathologist authorized by the Japanese Society of Pathology (YS), and classification of staging was done according to the Union for International Cancer Control (UICC) TNM staging system. The clinical and histopathological characteristics of the prostate cancer patients are summarized in Table 1. Written informed consent was obtained from all patients. This study was approved by the Ethics Committee of Tohoku University School of Medicine under the accession numbers of 2015-1-475 and 2015-1-476.

### 2.11. Laser Microdissection (LMD) and Methylation-Specific PCR (MSP)

FFPE sections (10 *μ*m thick) were prepared and mounted on 2.0 *μ*m thick PEN Membrane slides (MicroDissect GmbH, Herborn, Germany). The sections were deparaffinized, and then one section was stained with HE to evaluate the morphologic quality required for accurate microdissection. Other sections were stained with hematoxylin to recognize nuclei. After careful drying, cancerous and noncancerous prostate cells (~15 mm^2^) were separately collected into the caps of 200 *μ*l PCR tubes using Leica LMD7000 (Leica Microsystems GmbH, Wetzlar, Germany).

DNAs from microdissected FFPE samples were directly bisulfite-treated using EpiTect Fast DNA Bisulfite Kit (Qiagen). MSP analyses for *PYCARD* were performed with the primers described in Table S1. PCR was carried out for 40 cycles of 30 s at 94°C, 30 s at 68°C (unmethylated primers) or 30 s at 70°C (methylated primers), and 30 s at 72°C. The methylated and unmethylated band intensities were quantified by ImageJ software (National Institutes of Health, Bethesda, Maryland, USA) [27], and each methylated-to-unmethylated ratio was calculated; a twofold or higher level in tumor compared with the corresponding normal sample was defined as tumor-specific methylation. Each PCR product was loaded onto 4% agarose gels and visualized by ethidium bromide staining.

### 2.12. Immunohistochemistry

Four-micrometer slide sections were prepared, deparaffinized in xylene, and dehydrated in ethanol. Immunohistochemistry was performed as described before [28] using rabbit anti-human TMS1 antibody (EU107, 1 : 1000). Immunoreactivity was evaluated by two pathologists authorized by the Japanese Society of Pathology (YS and AG) without any information about the patients. In this study, stained tumor cells with 10% or more and 50% or more immunoreactivity in their cytoplasm or nuclei were classified as positive (+) and doubly positive (++), respectively; tumors with lower percentages were classified as negative (-).

### 2.13. Statistical Analysis

Fisher's exact test was used for associations between methylation status or immunostaining of *PYCARD* and clinicopathological parameters. A two-tailed Student *t*-test was used for associations between methylation status or immunostaining of PYCARD and PSA. Kaplan-Meier analysis and log-rank test were used to assess the difference in biochemical recurrence-free survival between patients with and without tumor-specific *PYCARD* methylation or PYCARD protein expression. *P* values of less than 0.05 were considered significant. All analyses were performed using JMP (SAS Institute, Cary, NC, USA).

## 3. Results

### 3.1. NF*κ*B (AD)-MBD Induction Significantly Suppresses Cell Growth by Inducing Apoptosis

To explore phenotypic alterations by reactivating hypermethylation-mediated inactivated genes, we first constructed tetracycline-regulated NF*κ*B (AD)-MBD-inducible cell lines using prostate cancer cell line LNCaP and a two-step procedure. First, pcDNA6/TR was introduced to establish two Tet repressor cells termed TR9 and TR15. Then, pcDNA4/TO/NF*κ*B (AD)-MBD (FLAG tag at the N-terminus) was introduced to establish two stable cell lines termed TR9_MeTA14 and TR15_MeTA5. As indicated in Figure S1, immunoblot analyses using anti-FLAG antibody indicated that tetracycline-induced NF*κ*B (AD)-MBD protein expression in both TR9_MeTA14 and TR15_MeTA5 cells was detected as late as 6 days after the induction. In other words, the induced MeTA is effective for at least 6 days.

**Figure 1:**
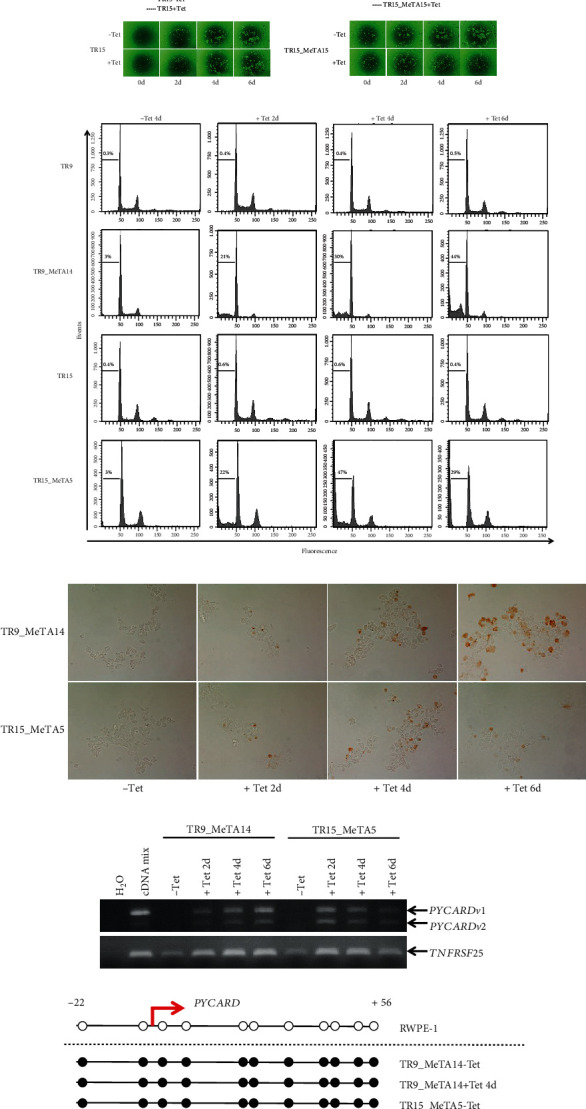
Effects of NF*κ*B (AD)-MBD induction on cell proliferation, apoptosis, and *PYCARD* upregulation. (a) Results of cell proliferation assays for LNCaP-derived TR9, TR9-MeTA14, TR15, and TR15-MeTA5. ∗ and ∗∗∗ denote *P* < 0.05 and *P* < 0.001, respectively. The photos below show the light microscopic appearance (40x magnification). Results of flow cytometry (b) and TUNEL ((c), 200x magnification) assays. (d) Results of RT-PCR analysis of *PYCARD* and *TNFRSF25* genes. (e) Methylation statuses obtained from bisulfite sequencing of *PYCARD* and *TNFRSF25* promoter regions are schematically shown. Closed and open circles indicate the methylated and unmethylated CpG sites, respectively. Red arrows indicate the transcriptional start site (TSS) at position +1. Incidental genetic alterations were found in LNCaP, in *TNFRSF25*, a G to A transition at position -64 (indicated by open triangles) and two deletions (indicated by closed triangles), one between +10 and +13 (4 bp) and the other between +46 and +53 (8 bp). These alterations contain one CpG site.

Global reactivation of hypermethylated genes may affect the growth of cancer cells. We next used an alamarBlue assay to examine the cell proliferation ability of our established LNCaP-derived cells in response to NF*κ*B (AD)-MBD induction (Figure 1(a)). Tet repressor-expressing parental cell lines, TR9 and TR15, did not show any growth differences irrespective of tetracycline treatment. On the other hand, NF*κ*B (AD)-MBD-inducible cell lines TR9_MeTA14 and TR15_MeTA5 showed significant growth suppression between 4 and 6 days after tetracycline addition. Interestingly, we observed cell shrinkage and pyknosis only in tetracycline-treated TR9_MeTA14 and TR15_MeTA5 cells (data not shown); these characteristics are typical of apoptotic cells. We then performed flow cytometry analyses as shown in Figure 1(b); the sub-G1 fractions in both TR9 and TR15 did not change before and after tetracycline addition, whereas the sub-G1 fractions in both TR9_MeTA14 and TR15_MeTA5 cells increased after tetracycline addition and gradually increased further over time, indicating that the G1, S, and G2/M phases of these cells gradually decreased over time. TUNEL assay was added and supported induction of apoptosis as indicated by results with FACS (Figure 1(c)). Some specific and strong signals representing apoptotic cells were observed only in cells treated with tetracycline: no such observations were evident in those without tetracycline. These results suggested that apoptosis and consequent growth suppression were induced by a NF*κ*B (AD)-MBD fusion protein. In other words, MeTA induced apoptosis in LNCaP.

### 3.2. NF*κ*B (AD)-MBD Induction Upregulates the Expression of Some Apoptosis-Inducing Genes

We then explored hypermethylated genes involved in apoptosis by microarray coupled with MeTA, termed MeTA-array [19]. TR9_MeTA14 and TR15_MeTA5 cells with or without tetracycline were used, and the results are summarized in Table 2. Five candidate genes were selected by the following criteria: (1) twofold or more upregulation by tetracycline induction in both TR9_MeTA14 and TR15_MeTA5 cells and (2) genes known to be involved in apoptosis induction. All five genes contained CpG islands (CGIs) within ±1000 bp of the transcription start site (TSS), suggesting that promoter regions of these genes may represent targets for MeTA. Because *PYCARD* and *TNFRSF25* were upregulated 10-fold or more in both TR9_MeTA14 and TR15_MeTA5 cells, we further analyzed these two genes. As shown in Figure 1(d), RT-PCR analyses demonstrated that both *PYCARD* and *TNFRSF25* were in fact upregulated in the presence of tetracycline. *PYCARD* has two splicing variants, *PYCARDv1* and *PYCARDv2*, the latter harboring an in-frame deletion of exon 2 encoding a segment of 19 amino acids sharing the same promoter. Both *PYCARDv1* and *PYCARDv2* contain functional PYD and CARD domains. These two *PYCARD* variants were upregulated similarly by tetracycline addition. Genomic bisulfite sequencing also confirmed hypermethylated promoters in the *PYCARD* and *TNFRSF25* enes (Figure S2) regardless of tetracycline treatment in both TR9_MeTA14 and TR15_MeTA5 cells. These sequencing results are schematically summarized in Figure 1(e). It is notable that the promoter region of *PYCARD* is unmethylated in RWPE-1, a nontumorigenic prostate epithelial cell line, but *TNFRSF25* showed hypermethylation. Based on these results, we focused on *PYCARD* for further characterization.

### 3.3. Tumor-Specific Hypermethylation of *PYCARD* Promoter Was Frequent in Prostate Cancers with Gleason Score ≥ 7

**Figure 2:**
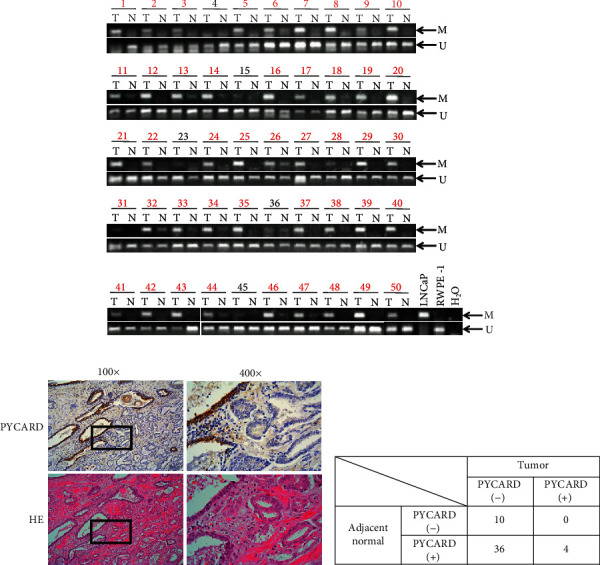
MSP and immunohistochemical analyses of PYCARD in prostate cancer specimens. (a) MSP analyses of *PYCARD* in 50 pairs of primary prostate tumors (T) and normal prostate epithelia (N). M and U indicate methylated- and unmethylated-specific PCR products, respectively. Red numbers indicate patients with tumor specific methylation. (b) Representative results of immunohistochemistry with HE staining in case no. 24. (c) Summary of immunohistochemical analyses. Results of individual patients are shown in Table S2.

We next analyzed methylation statuses in the promoter region of *PYCARD* in primary prostate cancer specimens. Results using 50 paired DNAs from cancerous and corresponding normal prostate tissues are shown in Figure 2(a) and summarized in Table S2. Aberrant methylation of *PYCARD* was detected in a tumor-specific manner in 90% (45/50); these results suggest that the aberrant hypermethylation of *PYCARD* promoter is frequent in primary prostate carcinomas. Therefore, the relationships between *PYCARD* promoter methylation and clinicopathological parameters such as Gleason score, T stages, lymphovascular invasion, and PSA value were analyzed (Table 1). Interestingly, methylation status of *PYCARD* promoter was significantly associated with Gleason score (*P* = 0.0063) or Grade Group (*P* = 0.002): prostate cancers with Gleason score ≥ 7 (Grade Group ≥ 2) but not Gleason score 6 (Grade Group 1) showed tumor-specific hypermethylation (Gleason score ≥ 7: 96%, 44/46, Gleason score 6: 25%, 1/4). We then divided patients with Gleason score 7 into Gleason score 3 + 4 (Grade Group 2) and Gleason score 4 + 3 (Grade Group 3) because patients with Gleason score 3 + 4 have been known to have a better prognosis than those with Gleason score 4 + 3 among patients with Gleason score 7 [29]. We examined the association between Grade Group 3 and 2 in *PYCARD* methylation statuses, but no significant associations were found (*P* = 0.9226). None of the patients died after the surgery. Therefore, the survival rate was 100% regardless of the *PYCARD* gene expression. In addition, *PYCARD* methylation did not show any relationships with PSA recurrence-free survival (Figure S3).

### 3.4. Immunohistochemical Analysis Shows the Loss of PYCARD Protein in Most of the Primary Prostate Cancers

We first analyzed the expression of PYCARD protein in a normal prostate epithelial cell line RWPE-1 and three prostate cancer cell lines, LNCaP, DU-145, and PC-3 (Figure S4). RWPE-1 showed a strong band of PYCARD, whereas LNCaP and DU-145 cells lost PYCARD protein expression and PC-3 showed a faint band. These results indicated that PYCARD expression is suppressed in prostate cancer cell lines. Therefore, we next performed immunohistochemical analyses of FFPE sections from 50 prostate cancer patients using anti-PYCARD antibody to see PYCARD protein expression. Results are summarized in Table S2. In most of the primary prostate cancer specimens (72%, 36/50), PYCARD protein was expressed in normal epithelial and/or basal cells, but not in tumor cells. Four patients showed positive immunostaining in both cancerous and normal cells, but normal cells were stained more intensely than tumor cells in 3 of 4 cases. Immunostaining of PYCARD in the remaining 10 prostate cancer specimens was negative for both normal and tumor cells. Representative results of immunohistochemical and HE stainings are shown in Figure 2(b), and the obtained results are summarized in Figure 2(c). These results indicate that PYCARD expression is strongly suppressed in tumor cells in most cases, but the status of *PYCARD* promoter hypermethylation is not completely consistent with the status of PYCARD expression. This status is probably regulated not only by aberrant DNA methylation but also by other unknown mechanisms. Furthermore, we analyzed the relationships between PYCARD expression and clinicopathological parameters, but we could not observe any associations (Table 1).

### 3.5. Role of PYCARD as an Apoptosis Inducible Factor

**Figure 3:**
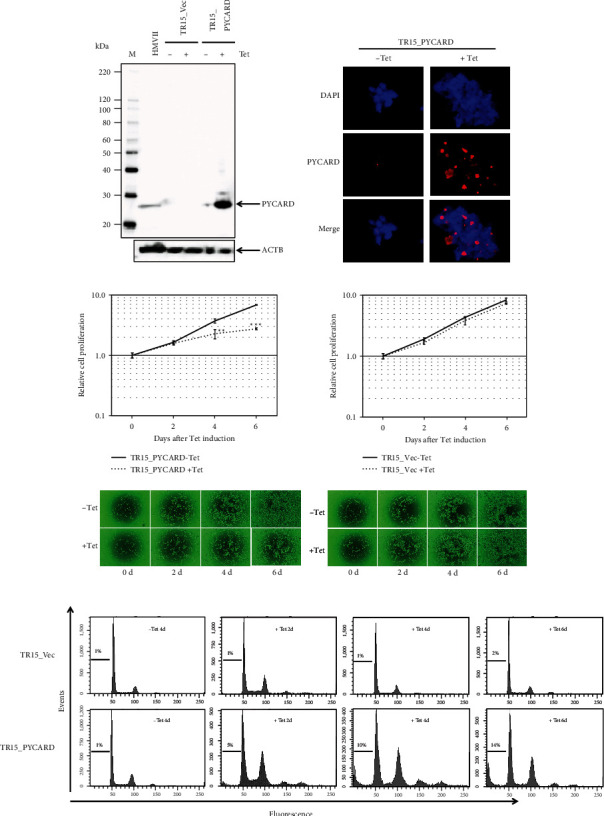
PYCARD reexpression induces apoptosis in LNCaP cells. (a) Immunoblotting analysis of PYCARD protein using anti-PYCARD antibody in LNCaP-derived cell lines. HMV-II is a human melanoma cell line used as a positive control. ACTB was used as an endogenous control. (b) Immunofluorescence staining of PYCARD (630x magnification). (c) Effects of PYCARD induction on TR15_PYCARD cell proliferation. ∗∗ and ∗∗∗ denote *P* < 0.01 and *P* < 0.001, respectively. Light microscopic appearances (40x magnification) of these cells are shown below. (d) Results of flow cytometry.

In order to investigate the biological significance of PYCARD in prostate cancer cells, we first transfected the *PYCARDv1*-expressing vector into TR15 to produce tetracycline-regulated PYCARDv1-inducible cells. A mixture of stably transfected cell lines termed TR15_PYCARD was established. Representative results of immunoblot and immunofluorescence analyses using this TR15_PYCARD at day 2 are shown in Figures 3(a) and 3(b). Expressed PYCARD protein forms speck-like protein complexes. Then, alamarBlue assays with or without tetracycline induction using TR15_PYCARD were performed (Figure 3(c)); induction of *PYCARD* caused significant growth suppression at days 4 and 6 after tetracycline addition. In contrast, empty-vector stably transfected TR15 cell lines termed TR15_Vec did not cause any growth suppression irrespective of tetracycline treatment (Figure 3(c)). We next performed FACS analysis of TR15_PYCARD to see whether the growth suppression by *PYCARD* induction affects the cell cycle. Our results of flow cytometry analyses in TR15_PYCARD are shown in Figure 3(d); sub-G1 fractions in TR15_PYCARD increased as early as two days after tetracycline addition and gradually increased further over time, but no such changes were observed in TR15_Vec. We also established a *PYCARDv2*-inducible stably transfected cell line mixture and performed the same experiments; the results did not essentially reveal any differences from those by *PYCARDv1-*transfected TR15_PYCARD (data not shown).

## 4. Discussion

Apoptosis-mediated programmed cell death serves as a natural barrier to initiation and progression of malignant transformation. Thus, acquisition of the ability to escape from these mechanisms is one of the major cancer hallmarks [1]. Tumor cells use a variety of strategies to limit or circumvent apoptosis. They may achieve this by increasing antiapoptotic regulators and survival signals, by decreasing proapoptotic factors, or by evading the ligand-induced death pathway. The most common tactic is the impairment of TP53 function; roughly half of the cancers have inactivating mutations of this tumor suppressor.

In this study, we searched for causative genes that induce apoptosis in the LNCaP prostate cancer cell line by MeTA-mediated global reactivation of hypermethylated genes; we identified five candidate apoptosis-inducing genes listed in Table 2. Among these, *PYCARD* was selected for further characterization. PYCARD, also known as ASC or TMS1, mediates assembly of large signaling complexes in the inflammatory and apoptotic signaling pathways *via* the activation of caspase [30]; aberrant hypermethylation in the promoter regions has been reported in prostate cancers and a number of tumors including cancers of the breast, lung, and kidney, as well as melanoma and glioblastoma [26, 31–37]. Virmani et al. reported that *PYCARD* methylation was seen in 41% (13 of 32) of small cell lung cancer tissues, 40% (28 of 70) of non-small-cell lung cancer tissues, and 32% (20 of 63) of breast cancer tissues [32]. On the other hand, *PYCARD* methylation was absent in nonmalignant lung tissues (0%, 0 of 18) and was rare in nonmalignant breast tissues (7%, 2 of 30). In general, a correlation was seen between *PYCARD* hypermethylation and reduced expression of the protein. Machida et al. used immunohistochemistry and reported that PYCARD expression was reduced in all lung cancer types (75%, 30 of 40) but not in 10 preinvasive lesions [33]. *PYCARD* methylation was particularly associated with later tumor stages of lung adenocarcinoma; only 14% (7 of 50) of stage I but 61% (19 of 31) of later-stage tumors showed methylation. Guan et al. immunohistochemically analyzed PYCARD expression and found that PYCARD expression was absent or reduced in 62.5% (20 of 32) of melanoma, whereas all 18 benign melanocytic nevi showed intense PYCARD expression [34]. Although we focused on *PYCARD* as a causative gene of apoptosis found by MeTA using LNCaP, MeTA also upregulates other candidate apoptosis-inducing factors as listed in Table 2. These other factors as well as PYCARD may collectively trigger the process of apoptosis.

In the 50 prostate cancer specimens we analyzed, *PYCARD* was hypermethylated in primary prostate cancers in a tumor-specific manner, consistent with previous studies [35–37]. Notably, we observed a significant difference in methylation frequency in association with Gleason score. Although Collard et al. [35] and Das et al. [36] reported cancer-specific hypermethylation of the *PYCARD* promoter in 65.5% (38/58) or 63.6% (42/66) of cases, these studies did not find any relationships between the methylation status of *PYCARD* and Gleason score. In our study, however, the great majority of cancer specimens (90%, 45/50) showed hypermethylation of the *PYCARD* promoter. In addition, we observed a significant association between hypermethylation of the *PYCARD* promoter and a Gleason score of 7 or higher (Gleason score ≤ 6: 25%, 1/4, ≥7: 96%, 44/46). Although the sample size of Gleason score ≤ 6 samples was limited, we think this finding is important. Further analyses will give us additional useful information for clinical management of patients with prostate cancer.

Although *PYCARD* hypermethylation was frequently associated with loss of PYCARD protein in prostate cancer specimens as shown in Figure 2(c) and Table S2, *PYCARD* methylation level is not always associated with PYCARD expression. Especially, in this study, loss of PYCARD protein was seen in normal epithelial and/or basal cells in 10 cases even though methylation levels of *PYCARD* promoter were extremely low. Similar relationships between *PYCARD*promoter methylation and expression have also been observed in primary melanomas [34] and glioblastomas [26]. There are several possibilities to explain these results. First, even though *PYCARD* promoter was unmethylated, PYCARD protein expression level may be determined by the degree of activation of transcription factors and the local microenvironment. In fact, PYCARD is known to be upregulated by cytokines such as tumor necrosis factor-*α* and interleukin- (IL-) 1*β* [38, 39]. Second, histone modification and chromatin remodeling in addition to *PYCARD* methylation may contribute to PYCARD protein expression. In this context, histone H4 lysine 16 acetylation (H4K16Ac) has been suggested to be important to maintain *PYCARD* gene activity [40].

It has been reported that *PYCARD* hypermethylation was identified not only in prostate cancer but also in prostate cancer precursor lesions called high-grade prostatic intraepithelial neoplasia (HGPIN) [35, 37]. Promoter hypermethylation of *PYCARD* appears to arise in an early stage in the pathogenesis of prostate carcinogenesis, and methylation levels progressively increase. Because tumor-specific *PYCARD* methylation was not frequent in Gleason score 6 specimens, analyses of *PYCARD* methylation in HGPIN should add further valuable information to clarify the role of *PYCARD* methylation in prostatic tumorigenesis. In addition, Alumkal et al. have shown that promoter methylation of *CDH13* alone or in combination with *PYCARD* independently associates with an increased risk of biochemical recurrence after radical prostatectomy [41]. All of these studies suggest that promoter hypermethylation of *PYCARD* can be potentially a biomarker in the clinical setting. At present, the measurement of PSA has been widely used as a blood test for earlier prostate cancer detection, but the proper use of this test is still controversial [42]. Because PSA is expressed in normal cells as well as in tumor cells, truly tumor-specific biomarker(s) responsible for the initiation and progression of prostate cancer are needed; preferably, it should be detectable by liquid biopsy. *PYCARD* is one of such candidates for prostate cancer diagnosis; further analyses are needed to clarify its role and utility value.

## 5. Conclusion

Using the methyl-CpG targeted transcriptional activation (MeTA) method, we identified apoptosis-inducing *PYCARD* as a DNA hypermethylation-mediated silencing gene, particularly in prostate cancer with Gleason score ≥ 7. Our present results suggest that promoter hypermethylation of *PYCARD* can be potentially a tumor-specific biomarker in the clinical setting of prostate cancer.

## Figures and Tables

**Table 1:** Relationships between *PYCARD* methylation or immunostaining and clinicopathological parameters.

Characteristics	Total (*n*)	Promoter methylation		*P* value	Immunostaining (T)		*P* value	Immunostaining (N)		*P* value
		Yes	No		Yes	No		Yes	No	
Total		45	5		4	46		40	10	
Gleason score (Grade Group)				0.0063 (0.002)			0.7726 (0.9134)			0.1759 (0.1463)
6 (group 1)	4	1	3		0	4		2	2	
7 (group 2)	20	20	0		2	18		17	3	
7 (group 3)	15	13	2		1	14		10	5	
8 (group 4)	5	5	0		0	5		5	0	
9 (group 5)	6	6	0		1	5		6	0	
Stage				1			1			0.2818
pT2≥	31	28	3		3	28		23	8	
pT3≤	19	17	2		1	18		17	2	
Surgical margin				1			1			0.4627
Yes	18	17	1		1	17		13	5	
No	32	28	4		3	29		27	5	
Lymphovascular invasion				1			1			0.3193
Yes	7	7	0		0	7		7	0	
No	43	38	5		4	39		33	10	
Biochemical recurrence^a^				0.3154			1			0.2326
Yes	12	12	0		1	11		8	4	
No	37	32	5		3	34		31	6	
Age (years), median, range	62 (51-78)	62 (51-78)	64 (54-74)		61.5 (57-69)	62 (51-78)		61.5 (51-78)	63.5 (54-74)	
PSA (ng/ml), median, range	7.6 (3.8-50)	8.1 (3.8-50.0)	7.1 (4.2-9.4)	0.3479	7.3 (5.7-10.8)	7.6 (3.8-50.0)	0.1276	8.0 (3.8-19.6)	6.8 (4.2-50)	0.2412

Statistical analysis was performed by using JMP. Fisher's exact test was used for associations between methylation status or immunostaining of PYCARD and clinicopathological parameters. A two-tailed Student *t*-test was used for associations between methylation status or immunostaining of PYCARD and PSA. ^a^Case no. 37 was excluded from this analysis because postoperative PSA levels did not drop to undetectable levels (0.2 ng/ml).

**Table 2:** Apoptosis-inducing genes upregulated after MeTA induction.

Gene of interest	Fold change		Existence of CGI in TSS ±1000 bp
	TR9 MeTA14	TR15 MeTA5	
*PYCARD*	175.8	64.8	Positive
*TNFRSF25*	17.6	12.3	Positive
*HRK*	2.6	6.1	Positive
*BIK*	3.3	2.8	Positive
*CIDEA*	2.2	2.3	Positive

## Data Availability

The data used to support the findings of this study are available in the GEO database (GSE74743) or from the corresponding author upon request.

## Supplementary Materials

Supplementary 1 Figure S1: NF*κ*B (AD)-MBD induction by tetracycline addition.

Supplementary 2 Figure S2: Representative bisulfite sequencing results of PYCARD (left) and TNFRSF25 (right) promoter regions.

Supplementary 3 Figure S3: The relationship between PSA recurrence-free rate and PYCARD expression or PYCARD tumor-specific methylation.

Supplementary 4 Figure S4: PYCARD is expressed in a normal prostate epithelial cell line but is rarely expressed in three prostate cancer cell lines.

Supplementary 5 Table S1: Nucleotide sequences of primers and probes used in this study.

Supplementary 6 Table S2: Results of DNA methylation and immunostaining analyses of PYCARD.
