# Supplementary material for: Aberrant Hypermethylation-Mediated Suppression of PYCARD Is Extremely Frequent in Prostate Cancer with Gleason Score ≥ 7
Source: Dis Markers. 2021 Feb 4;2021:8858905. doi: 10.1155/2021/8858905 (PMC7881737; doi:10.1155/2021/8858905)
Supplement: Supplementary 1 — Figure S1: NFκB (AD)-MBD induction by tetracycline addition. [file 8858905.f1.pdf]

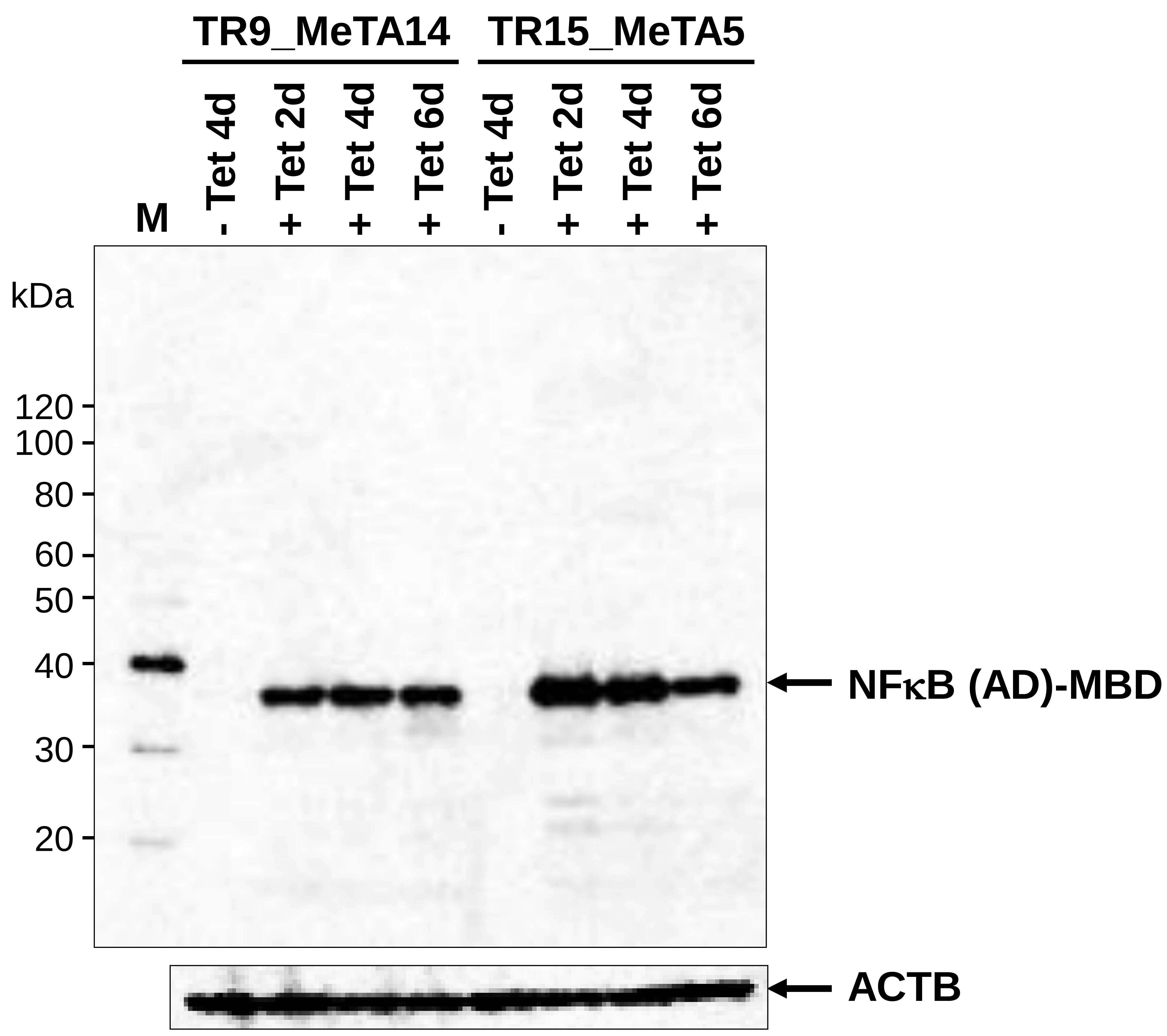

Figure S1. NFκB (AD)-MBD induction by tetracycline addition. Immunoblotting analysis of NFκB (AD)-MBD protein using anti-FLAG antibody in LNCaP-derived cell lines. +Tet and -Tet denote with and without tetracycline treatment, respectively. NFκB (AD)-MBD protein expressions were observed at days 2, 4, and 6 after tetracycline treatment in both TR9_MeTA14 and TR15_MeTA5 cells. Lane M contained MagicMark XP Standard used for protein size estimation. ACTB was used as the endogenous control.
