# Supplementary material for: Aberrant Hypermethylation-Mediated Suppression of PYCARD Is Extremely Frequent in Prostate Cancer with Gleason Score ≥ 7
Source: Dis Markers. 2021 Feb 4;2021:8858905. doi: 10.1155/2021/8858905 (PMC7881737; doi:10.1155/2021/8858905)
Supplement: Supplementary 2 — Figure S2: Representative bisulfite sequencing results of PYCARD (left) and TNFRSF25 (right) promoter regions. [file 8858905.f2.pdf]

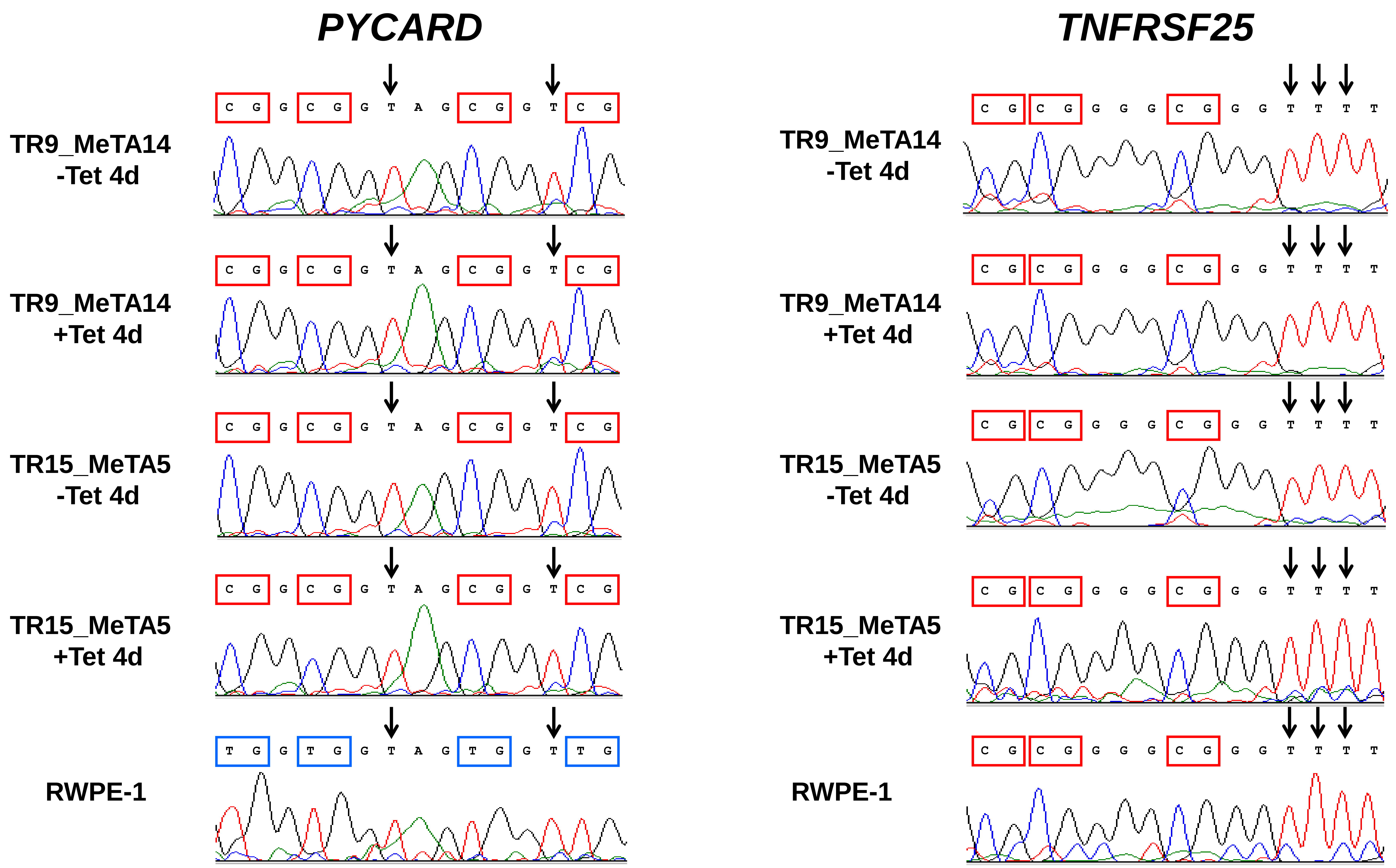

Figure S2. Representative bisulfite sequencing results of *PYCARD* (left) and *TNFRSF25* (right) promoter regions. *PYCARD* promoter region was highly methylated in LNCaP-derived prostate cancer cells but not in RWPE-1 normal prostate epithelial cells. Genomic DNAs were treated with sodium bisulfite and the promoter CGIs of *PYCARD* were sequenced. *TNFRSF25* promoter region was highly methylated both in LNCaP-derived prostate cancer cells and in RWPE-1 normal prostate epithelial cells. Arrows indicate the positions of cytosine residue converted by sodium bisulfite. Methylated CpG sites are boxed with red, and unmethylated CpG sites are boxed with blue.
