# Supplementary material for: Aberrant Hypermethylation-Mediated Suppression of PYCARD Is Extremely Frequent in Prostate Cancer with Gleason Score ≥ 7
Source: Dis Markers. 2021 Feb 4;2021:8858905. doi: 10.1155/2021/8858905 (PMC7881737; doi:10.1155/2021/8858905)
Supplement: Supplementary 3 — Figure S3: The relationship between PSA recurrence-free rate and PYCARD expression or PYCARD tumor-specific methylation. [file 8858905.f3.pdf]

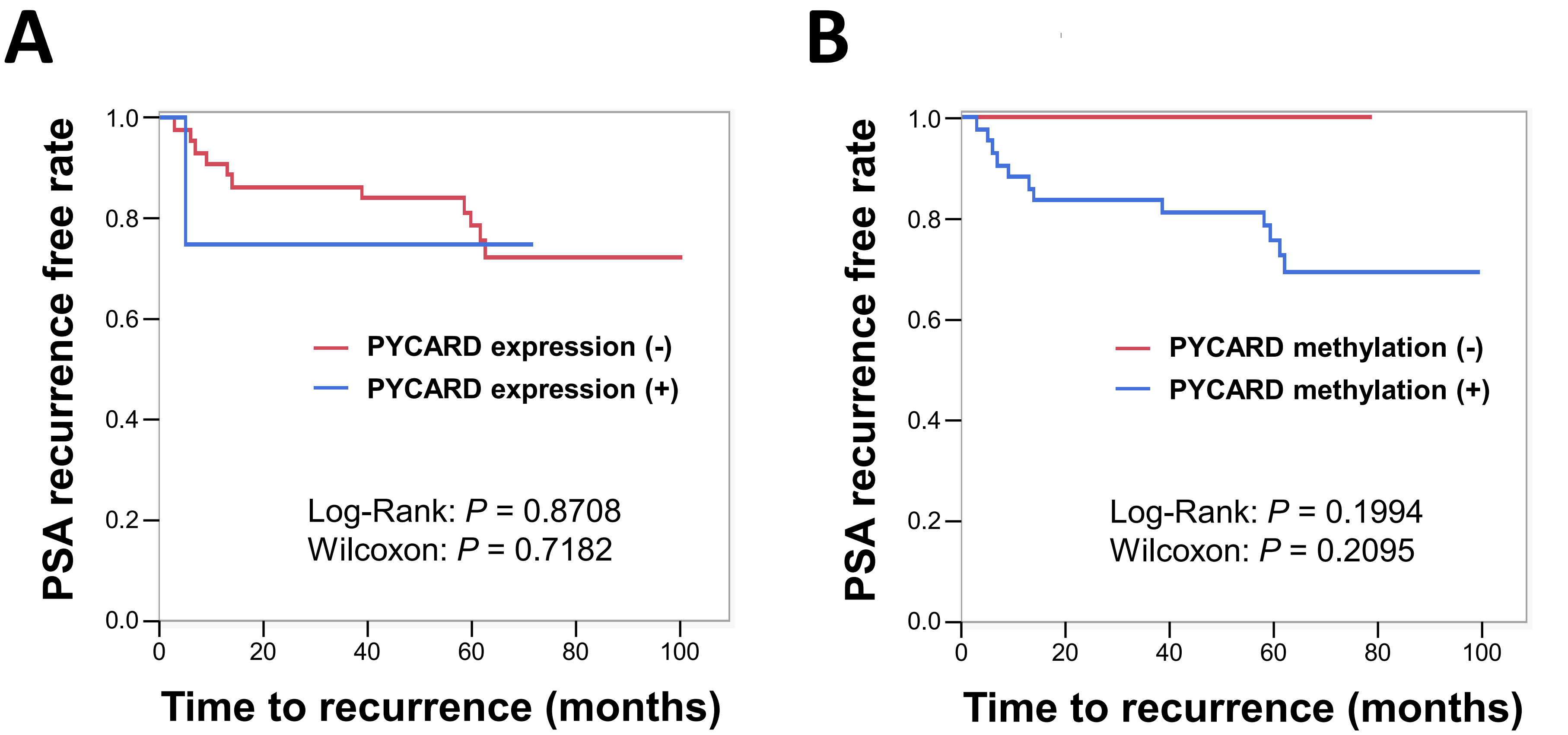

Figure S3. The relationship between PSA recurrence free rate and *PYCARD* expression or *PYCARD* tumor-specific methylation.

Kaplan-Meier analysis using 49 prostate cancer patients did not show any relationships between PSA recurrence free rate and *PYCARD* expression (A) or *PYCARD* tumor-specific methylation (B). Postoperative PSA levels in case #37 did not drop to undetectable levels (0.2 ng/ml). Therefore, we excluded this case from Kaplan-Meier analysis.
