# Supplementary material for: Aberrant Hypermethylation-Mediated Suppression of PYCARD Is Extremely Frequent in Prostate Cancer with Gleason Score ≥ 7
Source: Dis Markers. 2021 Feb 4;2021:8858905. doi: 10.1155/2021/8858905 (PMC7881737; doi:10.1155/2021/8858905)
Supplement: Supplementary 4 — Figure S4: PYCARD is expressed in a normal prostate epithelial cell line but is rarely expressed in three prostate cancer cell lines. [file 8858905.f4.pdf]

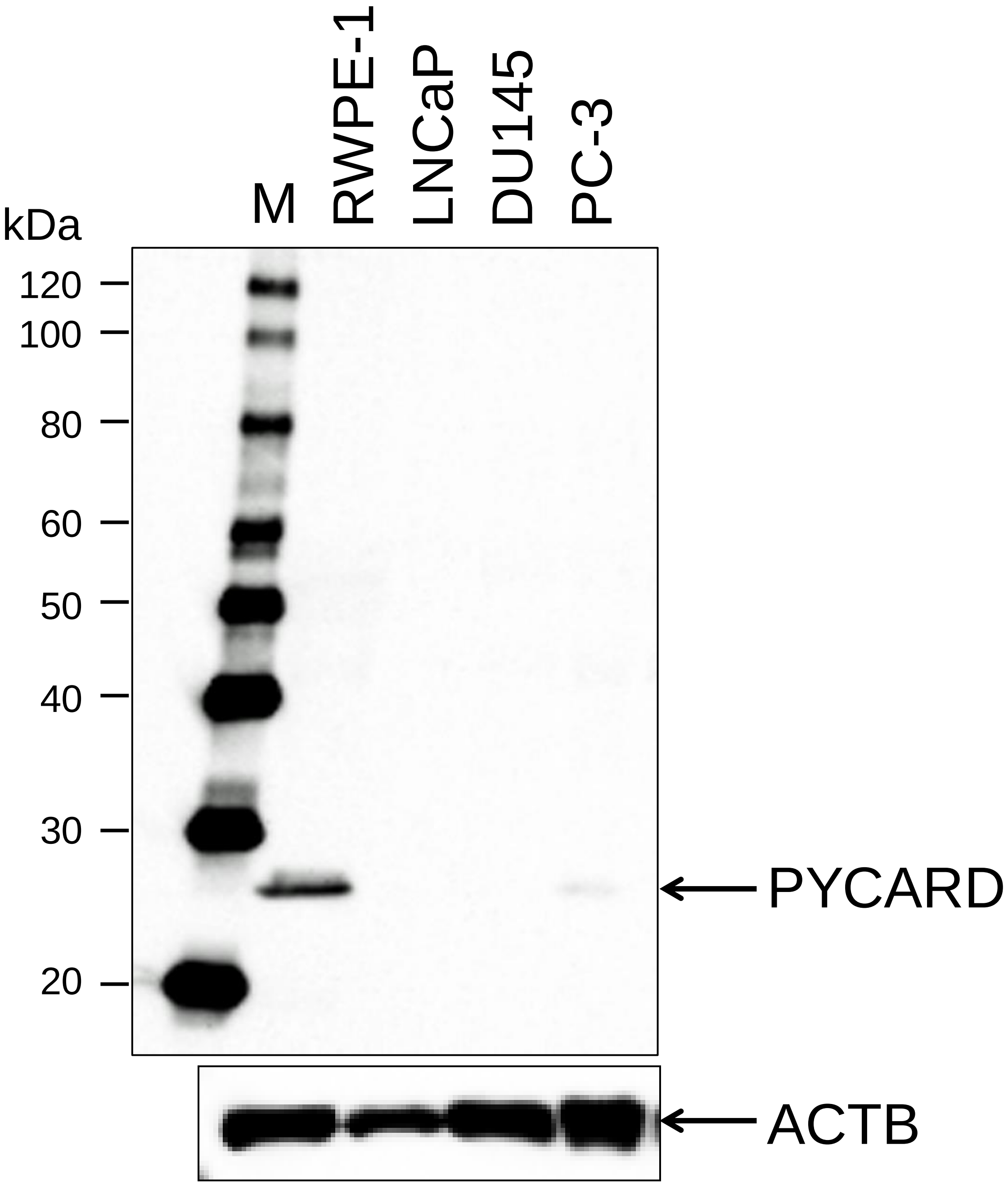

Figure S4. PYCARD is expressed in a normal prostate epithelial cell line but is rarely expressed in three prostate cancer cell lines.

Immunoblotting analysis of PYCARD protein in a normal prostate epithelial cell line RWPE-1 and three prostate cancer cell lines, LNCaP, DU145, and PC-3. Lane M contained MagicMark XP Standard used for protein size estimation. ACTB was used as the endogenous control.
