# Supplementary material for: Aberrant Hypermethylation-Mediated Suppression of PYCARD Is Extremely Frequent in Prostate Cancer with Gleason Score ≥ 7
Source: Dis Markers. 2021 Feb 4;2021:8858905. doi: 10.1155/2021/8858905 (PMC7881737; doi:10.1155/2021/8858905)
Supplement: Supplementary 5 — Table S1: Nucleotide sequences of primers and probes used in this study. [file 8858905.f5.pdf]

Table S1. Nucleotide sequences of primers and probes used in this study

Gene	Forward primer (5' to 3')	Reverse primer (5' to 3')	Annealing temp. (^{o}C)	Amplified region*	Size of product (bp)
Cloning					
PYCARDv1	GATAAGCTTGCCGCCACCATGGGGCGCGCGCG	GATGCTAGCTCAGCTCCGCTCCAGGTC	60	+71 to +658	615
PYCARDv2	GATAAGCTTGCCGCCACCATGGGGCGCGCGCG	GATGCTAGCTCAGCTCCGCTCCAGGTC	60	+71 to +601	558
RT-PCR					
PYCARD	CAGCTTCTACCTGGAGACC	CATCCAGCAGCCACTCAACG	62	+241 to +473	233
TNFRSF25	GGTCCTGGGACCAGTTGC	GTCCATCACGTCGTAGAGC	60	+1008 to +1135	128
*B2M***	GTGGAGCATTCAGACTTGTC	CCAAATGCGGCATCTTCAAAC	55	+265 to +455	191
Bisulfite Sequencing					
PYCARD-BS	GAGATTAGAGTGGGAGGAAGG	CCCCATAACTCCAAAATCCC	60	-43 to +76	119
TNFRSF25-BS	GAGTAGAGGGGGTATTTGGT	CAAAACTACCCCTAACCTCC	60	-91 to +162	253
MSP					
PYCARD MSP M	TTGTAGCGGGGTGAGCGGC	CCAACGCATCCAAAATAACGTCG	70	+27 to +107	81
PYCARD MSP U	GGTTGTAGTGGGGTGAGTGGT	AAATTCTCCAACACATCCAAAATAACAT	68	+25 to +114	90

* +1 indicates transcriptional start site.

** Ogawa et al. [23]
