# Supplementary material for: Aberrant Hypermethylation-Mediated Suppression of PYCARD Is Extremely Frequent in Prostate Cancer with Gleason Score ≥ 7
Source: Dis Markers. 2021 Feb 4;2021:8858905. doi: 10.1155/2021/8858905 (PMC7881737; doi:10.1155/2021/8858905)
Supplement: Supplementary 6 — Table S2: Results of DNA methylation and immunostaining analyses of PYCARD. [file 8858905.f6.pdf]

Table S2. Results of DNA methylation and immunostaining analyses of PYCARD

Patient No.	methylation analysis		immunostaining analysis			Gleason score	Grade Group	PSA recurrence	Time to recurrence (month)
	Tumor specific methylation	T/N[a]	Tumor	Normal (E)[b]	Normal (B)[c]				
1	P	5.8	N	N	P+	8	4		
2	P	2.5	N	N	P+	7	3		
3	P	2.4	N	P++	P++	7	2		
4	N	1.2	N	N	N	6	1		
5	P	3.6	N	N	N	7	2	+	7
6	P	2.0	P+	P+	P+	7	2		
7	P	3.0	N	P+	P+	7	2		
8	P	3.7	P+	P++	P++	7	2		
9	P	2.0	N	N	N	7	2		
10	P	2.8	N	P+	P+	7	2		
11	P	2.5	N	N	N	7	3	+	13
12	P	7.1	N	P++	P++	7	2	+	6
13	P	4.7	N	P+	P++	7	2		
14	P	8.8	N	P+	P++	7	2		
15	N	1.1	N	P+	P++	7	3		
16	P	7.2	N	P+	P++	8	4		
17	P	5.1	N	P+	P+	9	5	+	14
18	P	4.3	N	P+	P+	7	2		
19	P	6.8	N	N	N	7	2		
20	P	12.1	N	P+	P++	8	4		
21	P	6.4	N	P+	P+	7	3		
22	P	2.6	P+	P++	P++	7	3		
23	N	0.4	N	N	N	6	1		
24	P	4.4	N	P+	P++	7	2		
25	P	4.7	N	P+	P+	7	2		
26	P	2.1	N	P+	P+	7	2		
27	P	2.1	N	P+	P+	7	3		
28	P	2.1	N	P+	P+	7	2	+	59
29	P	4.9	N	P++	P++	7	3		
30	P	4.5	N	P++	P++	7	2		
31	P	4.0	N	P+	P++	7	3		
32	P	2.2	N	P++	P++	9	5		
33	P	3.6	N	P+	P+	7	2		
34	P	7.1	N	P+	P++	8	4	+	63
35	P	10.2	N	P+	P+	9	5		
36	N	0.9	N	P+	P++	6	1		
37	P	6.6	N	P+	P+	9	5		
38	P	5.7	N	P+	P++	8	4	+	9
39	P	59.7	N	P+	P++	9	5	+	60
40	P	9.3	N	N	P+	7	2		
41	P	2.6	N	P++	P++	6	1		
42	P	5.0	N	P+	P+	7	3		
43	P	48.9	P+	P++	P++	9	5	+	5
44	P	2.1	N	P++	P++	7	3		
45	N	1.4	N	N	N	7	3		
46	P	3.3	N	P+	P+	7	2		
47	P	2.9	N	N	N	7	3	+	39
48	P	5.5	N	N	N	7	3	+	62
49	P	6.1	N	N	P+	7	3	+	3
50	P	7.7	N	N	N	7	3		

[a]Ratio of signal intensities in tumor vs. normal tissues.

(E)[b] and (B)[c] represent epithelial and basal cells, respectively.
